# Supplementary material for: MetaRibo-Seq measures translation in microbiomes
Source: Nat Commun. 2020 Jun 29;11:3268. doi: 10.1038/s41467-020-17081-z (PMC7324362; doi:10.1038/s41467-020-17081-z)
Supplement: Supplementary file 10 — Supplementary Data 7 [file 41467_2020_17081_MOESM10_ESM.zip › File2/Confidence_High_Taxonomy/119363_out.krona.html]

Javascript must be enabled to view this page.

members
magnitude
magnitudeUnassigned
count
unassigned
taxon
rank

119363\_out

2

superkingdom
2
2

2
976
phylum

2
class
200643

order
171549
2

815
family
2

genus

SRS052027\_contig\_number\_17108
1
816
2

371601
species
1

SRS1041112\_contig\_number\_1925
